# Baicalin Protects Against 17α-Ethinylestradiol-Induced Cholestasis *via* the Sirtuin 1/Hepatic Nuclear Receptor-1α/Farnesoid X Receptor Pathway

**DOI:** 10.3389/fphar.2019.01685

**Published:** 2020-02-11

**Authors:** Jinyu Yang, Daochun Xiang, Dong Xiang, Wenxi He, Yanan Liu, Lulu Lan, Guodong Li, Chen Jiang, Xiuhua Ren, Dong Liu, Chengliang Zhang

**Affiliations:** ^1^Department of Pharmacy, Tongji Hospital Affiliated Tongji Medical College, Huazhong University of Science and Technology, Wuhan, China; ^2^The Central Hospital of Wuhan, Tongji Medical College, Huazhong University of Science and Technology, Wuhan, China; ^3^College of Pharmacy, Jilin University, Changchun, China

**Keywords:** baiclain, cholestasis, 17α-ethinylestradiol, sirtuin 1, hepatic nuclear receptor-1α, farnesoid X receptor

## Abstract

Estrogen-induced cholestasis (EIC) is characterized by impairment of bile flow and accumulated bile acids (BAs) in the liver, always along with the liver damage. Baicalin is a major flavonoid component of *Scutellaria baicalensis*, and has been used in the treatment of liver diseases for many years. However, the role of baicalin in EIC remains to be elucidated. In this study, we demonstrated that baicalin showed obvious hepatoprotective effects in EIC rats by reducing serum biomarkers and increasing the bile flow rate, as well as by alleviating liver histology and restoring the abnormal composition of hepatic BAs. In addition, baicalin protected against estrogen-induced liver injury by up-regulation of the expression of hepatic efflux transporters and down-regulation of hepatic uptake transporters. Furthermore, baicalin increased the expression of hepatic BA synthase (CYP27A1) and metabolic enzymes (Bal, Baat, Sult2a1) in EIC rats. We showed that baicalin significantly inhibited hepatic inflammatory responses in EIC rats through reducing elevated levels of TNF-α, IL-1β, IL-6, and NF-κB. Finally, we confirmed that baicalin maintains hepatic BA homeostasis and alleviates inflammation through sirtuin 1 (Sirt1)/hepatic nuclear receptor-1α (HNF-1α)/farnesoid X receptor (FXR) signaling pathway. Thus, baicalin protects against estrogen-induced cholestatic liver injury, and the underlying mechanism involved is related to activation of the Sirt1/HNF-1α/FXR signaling pathway.

## Introduction

Estrogen-induced cholestasis (EIC) is one of the most common liver diseases, and mainly occurs during pregnancy, oral contraceptives or hormone replacement therapy (Rezai et al., 2015). EIC is characterized by dysfunction of bile acid (BA) homeostasis, leading to BA accumulation in the liver, thereby resulting in cholestatic liver injury, and poor fetal outcomes (Pusl and Beuers, 2007; Dong et al., 2019). However, in the clinic, drug therapies and efficacy are limited for EIC patients. Currently, the first line treatment of cholestastic liver disease is ursodeoxycholic (UDCA) to which approximately 40% of patients have inadequate response (Woolbright and Jaeschke, 2016; El-Hawary et al., 2019). Therefore, it is of utmost importance to identify novel therapeutic compounds for the treatment of EIC.

**Figure 1:**
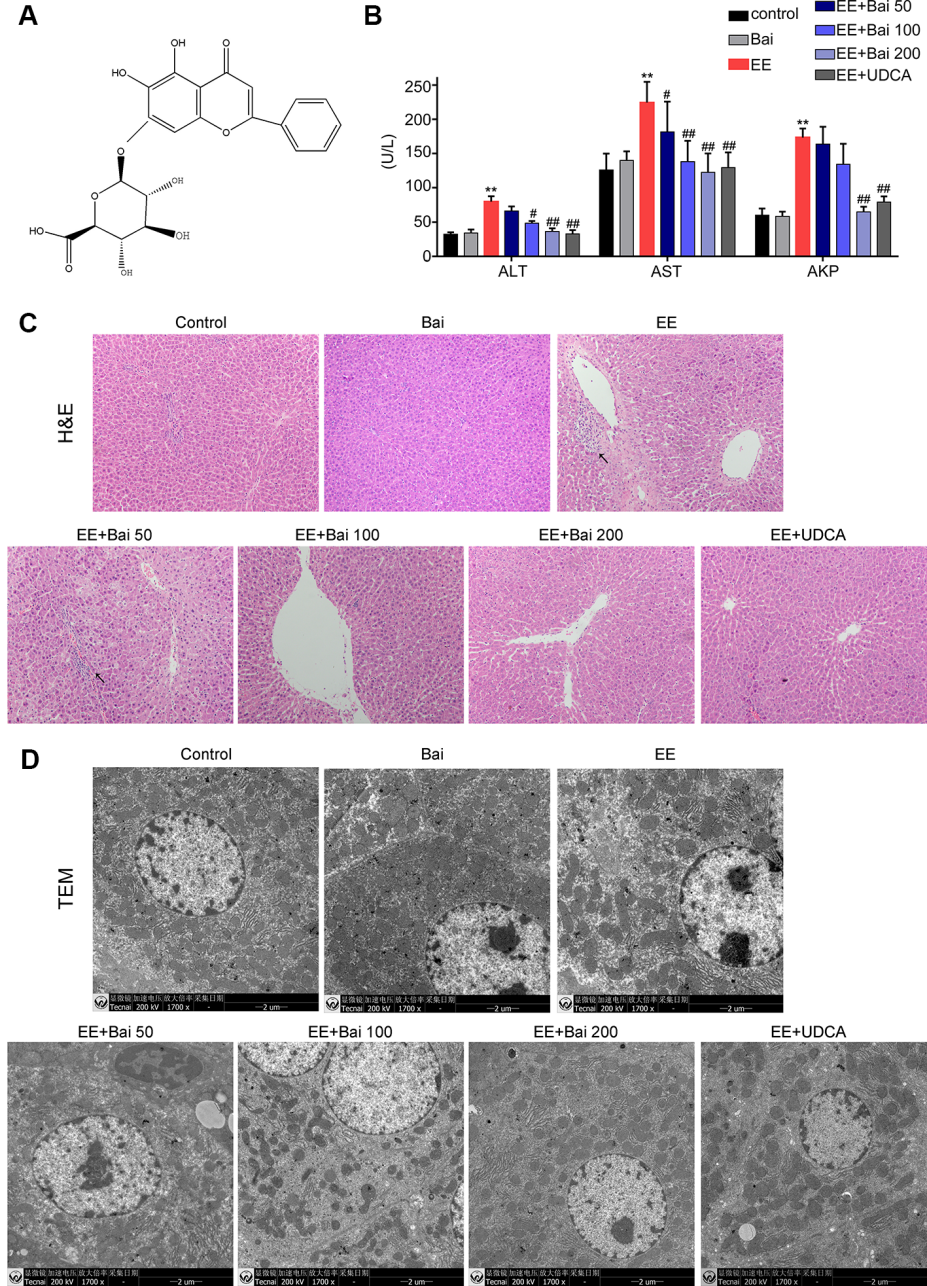
Hepatoprotective effects of baicalin against EE-induced hepatotoxicity. **(A)** The chemical structure of baicalin. **(B)** Serum aminotransferase (ALT), aspartate aminotransferase (AST), and AKP. **(C)** H&E stained liver sections showing the hepatic structure changes. Areas of inflammatory cells infiltration are indicated by arrows. **(D)** TEM images showing substructural changes of hepatocytes. **(D)** The bile flow rate in rats over 2 hours. Data are presented as the mean ± SD. (n = 6). **p < 0.01 versus the control group, ^#^p < 0.05 and ^##^p < 0.01 versus the EE group.

Presently, Chinese Traditional Medicines have gained growing interest as potential therapeutic drugs (Guo et al., 2009; Yao et al., 2018; Zhang J et al., 2018). *Scutellaria baicalensis* is the dried root from the perennial herb *Scutellaria baicalensis Georgi*, and has been widely used in traditional Chinese medicine for many years. Baicalin (**Figure 1A**) is the main component isolated from the raw extracts of *Scutellaria baicalensis*, and is a bioactive flavonoid that possesses multiple biological functions both *in vivo* and *in vitro*, including anti-inflammatory, anti-oxidation, and anti-obesity properties (Lixuan et al., 2010; He et al., 2017; Dai et al., 2018). It has previously been reported that baicalin can ameliorate experimental liver cholestasis caused by bile duct ligation (BDL) in mice (Shen et al., 2017). However, studies on the role of baicalin in 17α-ethinylestradiol (EE)-induced cholestasis are limited. In our previous study, we demonstrated the protective effects of baicalin on EIC rats, and explored the pharmacokinetic characteristics of baicalin (Zhang CL et al., 2018). However, the underlying mechanism of its protective effects on EE-induced cholestasis remains to be elucidated.

EE is a synthetic estrogen derivate that has been widely used in laboratory settings to explore the molecular mechanisms involved in EIC (Rodríguez-Garay, 2003). In experimental animals, EE treatment showed bile obstruction and hepatic toxic BA accumulation, which resulted in hepatotoxicity (Yamamoto et al., 2006). The main pathogenic causes included that EE treatment inhibited the expression of BA synthetic enzymes, metabolism enzymes, and BA transporters, thereby disturbing BA homeostasis that caused accumulation of toxic BAs in the liver (Xiang et al., 2019). In addition, inflammatory responses are also an important pathogenic factor in EE-induced liver injury (Yu et al., 2016; Xu et al., 2017; El-Hawary et al., 2019). BA homeostasis is tightly regulated by hepatic nuclear receptors, including farnesoid X receptor (FXR, NR1H4), vitamin D receptor (VDR, NR1I1), constitutive androstane receptor (CAR, NR1I3), and the pregnane X receptor (PXR, NR1I2) (Li and Chiang, 2013). Among them, FXR is one of the most important BA sensors in maintaining BA homeostasis through regulating BA synthesis, metabolism, and transport (Kuipers et al., 2004). It has also been shown that activation of FXR exerts anti-inflammatory effects in liver diseases (Zhang et al., 2017). However, FXR does not work alone (Kemper et al., 2009; Li et al., 2017) and is regulated by multiple signaling pathways (Zhang et al., 2004; Li et al., 2016; Yang et al., 2017).

Sirtuin 1 (Sirt1) is a nicotinamide adenine dinucleotide (NAD)^+^-dependent deacetylase, and has been considered a key sensor in regulating a wide range of metabolic processes by de-acetylating and activating the transcription of metabolic regulators, including peroxisome proliferator-activated receptor gamma, coactivator 1 alpha (PGC-1α), hepatic nuclear receptor (HNF)-1α, nuclear factor (NF)-κB, liver X receptor (LXR), and FXR, which are involved in lipid and glucose metabolism, inflammation, and BA homeostasis (Leibiger and Berggren, 2006; Li, 2013; Chang and Guarente, 2014). It is well known that Sirt1 is a key factor in regulating BA homeostasis through modulating the FXR signaling pathway (Kemper et al., 2009; Lee and Kemper, 2010). Sirt1 can modulate the expression and transactivation of FXR through the HNF-1α pathway (Kazgan et al., 2014). It has previously been reported that hepatic deletion of Sirt1 reduced FXR activation *via* HNF-1α signaling, which ultimately resulted in reduced BA transport and absorption (Purushotham et al., 2012). Moreover, activation of Sirt1/HNF-1α/FXR signaling can protect against estrogen-induced liver injury (Yu et al., 2016). Thus, activating FXR *via* the Sirt1/HNF-1α pathway may be a therapeutic strategy for treating cholestatic liver injury.

The purpose of the present study was to evaluate the hepatoprotective effects of baicalin on EE-induced cholestatic liver injury, and to explore the underlying mechanisms involved. Our findings demonstrated that the protective effect of baicalin in EIC rats was due to maintaining hepatic BA homeostasis and the anti-inflammatory effects *via* Sirt1/HNF-1α/FXR signaling pathway.

## Materials and Methods

### Chemicals and Regents

Baicalin (>98%) was purchased from Dalian Meilun Biotechnology Co., Ltd (Meilunbio, Dalian, China). EE was purchased from Sigma-Aldrich Co. (St. Louis, MO, USA). Standards for BAs for cholic acid (CA), deoxycholic acid (DCA), chenodeoxycholic acid (CDCA), β-muricholic acid (β-MCA), taurocholic acid (TCA), taurodeoxycholic acid (TDCA), tauromuricholic acid (T-MCA), taurochenodeoxycholic acid (TCDCA), glycocholic acid (GCA), glycoursodesoxycholic acid (GUDCA), glycohyocholic acid (GHDCA), glycochenodeoxycholic acid (GCDCA), taurohyocholic acid (THDCA), glycodeoxycholic acid (GDCA), and interior label d4-glycochenodeoxycholic acid (d4-GCDCA) were obtained from Steraloids (Newport, RI, USA).

Antibodies directed against FXR, CAR, PXR, VDR, cholesterol 7a-hydroxylase (CYP7A1), and sterol 27-hydroxylase (CYP27A1) were purchased from Absin Biochemical Company (Shanghai, China). Antibodies directed against Sirt1, HNF-1α, tumor necrosis factor α (TNF-α), interleukin 1 beta (IL-1β), IL-6, and NF-κB were purchased from Cell Signaling Technology (Beverly, MA, USA). An antibody against BSEP was obtained from Santa Cruz Biotechnology (CA, USA). And an antibody against MRP2 was obtained from Abcam (Cambridge, UK).

### Animals and Drug Treatments

Male Sprague Dawley (SD) rats (8 weeks, 200 ± 20 g) were obtained from the center of Experimental Animal of Tongji Medical School, Huazhong Science and Technology University (Hubei Wuhan, China). Animal experiments were conducted using the National Institutes of Health guide for the care and use of laboratory animals. All animal studies were approved by the Ethics Committee on Animals Experimentation of the Tongji Hospital (Tongji, China). All animals were housed in a temperature- and humidity-controlled environment under a constant 12-hour light-dark cycle and maintained on a standard laboratory diet and water that was provided ad libitum. After 1-week of acclimatization, animals were randomly divided into seven groups (n = 6) as follows: control group, baicalin-treated group, model (EE) group, baicalin-treated (50, 100, and 200 mg/kg) groups, and ursodesoxycholic acid (UDCA)-treated (40 mg/kg) groups. In brief, rats were subcutaneously injected with EE (5 mg/kg) or vehicle (propylene glycol) and orally administrated with baicalin (50, 100, or 200 mg/kg) and UDCA (40 mg/kg) or vehicle (10% sodium carboxymethylcellulose) once daily for five consecutive days. All rats were sacrificed on the 6th day after overnight fasting. Livers were removed and liver weights were recorded. Bile, blood, livers, and kidney samples were collected and stored for subsequent analyses.

### Serum Biochemical Analyses

Levels of serum alanine aminotransferase (ALT), alkaline phosphatase (AKP), aspartate aminotransferase (AST), total bile acid (TBA), total bilirubin (TBIL), and direct bilirubin (DBIL) were determined using commercial kits according to the manufacture’s guidelines (Jiancheng Bioengineering Institute, Nanjing, China).

### Bile Flow Rate Measurements

Using general anesthesia, rat bile was collected into PE10 polyethylene tubes. Bile was collected every 30 minutes for 2 hours while the rats were kept under anesthesia at room temperature. Bile volume was judged gravimetrically at a density of 1.0 g/ml.

### Histopathology

Liver samples were fixed in 10% neutral formalin, embedded in paraffin, cut into 6-μm sections, and stained with hematoxylin and eosin (H&E). Images to assess for structural changes were captured using a light microscope (OLYMPUS, Tokyo, Japan).

For transmission electron microscopy (TEM) observation of the liver, liver tissue sections were fixed in 3% glutaraldehyde solution for 2 hours, then fixed in 1% osmic acid for 1 hour, dehydrated in ethanol, and solidified by acetone gradient and embedded. Subsequently, ultrathin sections of 60 to 80 nm were cut. After staining sections with uranyl acetate-lead citrate, microstructural changes of the liver were observed under TEM (FEI Company, Tecnai G2, US).

### Analysis of BA in the Liver by Liquid Chromatography-Tandem Mass Spectrometry (LC-MS/MS)

BAs were extracted from liver samples and analyzed by high-performance liquid chromatography-tandem mass spectrometry as previously before (Xiang et al., 2019).

### Quantitative Real-Time PCR

Total RNA from rat liver tissue was extracted using TRIzol reagent (Invitrogen Life Technology, CA, USA). From each sample, 2 μg/μl of total RNA was reverse-transcribed into cDNA using PrimeScriptTM RT Marter Mix (Takara, Dalian, China). SYBR PCR Master Mix (Takara, Dalian, China) was used to quantify gene expression. Reports were read by using the ABI StepOne Plus system (Applied Biosystems, CA, USA). Internal β-actin was used as a standard to normalize the mRNA quantity. The primer sequences that were used for the tested genes are listed in **Supplementary Tables 1** and **2**.

### Western Blot Analysis

Liver proteins were collected by using RIPA lysis buffer (Beyotime Biotechnology, China), containing 1 mM phenylmethanesulfonyl fluoride, protease, and a phosphatase inhibitor cocktail. The protein concentration was determined by using a BCA protein assay reagent kit (Beyotime Biotechnology, China). Per sample, 30 μg protein was resolved using 8–12% SDS-PAGE, and transferred into PVDF membranes. Next, membranes were incubated with Tris-buffered saline, containing 5% nonfat dry milk for blocking purposes at room temperature for 1 hour. Then, membranes were incubated overnight at 4°C with primary antibodies directed against FXR, CAR, PXR, VDR, CYP7A1, CYP27A1, Na^+^-dependent taurocholate cotransport peptide (NTCP), bile salt export pump (BSEP), multidrug resistance-associated protein 2 (MRP2), Sirt1, HNF-1α, TNF-α, IL-1β, IL-6, and NF-κB. Subsequently, membranes were incubated with horseradish peroxidase (HRP)-conjugated antibodies at room temperature for 1 hour. Protein expression was visualized by an enhanced chemiluminescence (ECL) method (Absin, Shanghai, China).

### Immunohistochemistry

The protein expression levels of NTCP, BSEP, and MRP2 were also analyzed by immunohistochemistry. For immunohistochemically staining, paraffin sections were dewaxed in xylene and rehydrated in graded alcohol. Then, 10 mmol/citrate buffer (pH 6) was used for antigen retrieval under the microwave heating (20 minutes). To block the endogenous peroxidase activity, the sections were immersed in H_2_O_2_ (0.3%) for 25 minutes at room temperature and protect against light. Followed by blocking with 5% non-fat dry milk for 30 minutes. The sections were incubated with primary anti-NTCP, anti-BSEP, and anti-MRP2 overnight at 4°C. Sections were then incubated with a secondary horseradish peroxidase (HRP)-conjugated antibody for 50 minutes at RT and then developed by diaminobenzidine (DAB). The sections were stained with hematoxylin for 3 minutes. Sections were then dehydrated in a gradient alcohol and xylene solution and mounted in neutral gum. Last, images were taken using an Olympus microscope (Tokyo, Japan), and analyzed by Image-Pro Plus 6.0 software (Media Cybernetics, Inc., Rockville, MD, USA). The mean density was quantified by counting three different fields per section (200×).

### Cell Culture

HepG2 cells were cultured in Dulbecco’s Modified Eagle’s Medium (DMEM) medium supplemented with 10% FBS, 100 U/ml penicillin, and streptomycin in a environment containing 5% CO_2_ at 37°C. In brief, cells were plated in 6-well plates at a density of 3.8*105 per well. After overnight incubation, EE (10 μM), EE (0 μM) with baicalin (20 μM), EE (10 μM) with baicalin (40 μM), or EE (10 μM) with baicalin (80 μM) were added to the culture medium for 24 hours. Then, cells were harvested for quantitative real-time PCR and Western blot analysis.

### RNA Silencing of Sirt1 in HepG2 Cells

When HepG2 cells reached 70–90% confluency, cells were transfected with 200 nM shRNA targeting human sirt1 or a negative control shRNA using Lipofectamine^®^ 2000 reagent. Six hours later, fresh medium was added. After 24 hours of transfection, the medium was replaced. After 48 hours, EE (10 μM) and baicalin (80 μM) were added to the medium, and cells were cultured for 24 hours before they were harvested for further experiments.

### Statistical Analysis

All data were assessed using GraphPad Prism7 and presented as the mean ± SD. Significant differences between two groups were evaluated by Student’s t-test, and multiple comparisons were followed by one-way analysis. Values of p < 0.05 were considered statistically significant.

## Results

### Baicalin Protects Against EE-Induced Hepatotoxicity

Body weights of EE-treated rats were significantly decreased, and both liver weight as well as the liver index were markedly increased (p < 0.01) (**Table 1**). After treatment with baicalin 50, 100, and 200 mg/kg, no significant changes in body weight were observed. However, liver weight and the liver index were significantly decreased by baicalin treatment (**Table 1**). Serum biochemical indexes, such as ALT, AST, and AKP, which are indicators of hepatotoxicity, were significantly increased in EIC rats and were reduced by baicalin treatment in a dose-dependent manner (**Figure 1B**). UDCA, used as a positive control in our study, is well-known for cholestatic disease therapy, and markedly reduced liver weight, the liver index, and elevated serum biochemical indexes in EIC rats.

**Table 1:** Effects of baicalin on body weight, liver weight, and liver index of EE-induced rats (mean ± SD, n = 6).

Groups	Body weight (g)	Liver weight (g)	Liver index (%)
Control	257.75 ± 21.90	8.88 ± 3.29	3.41 ± 0.13
Baicalin	263.44 ± 30.12	8.06 ± 2.34	3.09 ± 0.16
EE	246.63 ± 20.61*	12.45 ± 3.94**	4.68 ± 0.08**
EE+Bai 50	249.38 ± 29.68	11.29 ± 2.34^#^	4.05 ± 0.19^#^
EE+Bai 100	237.88 ± 16.24	10.78 ± 1.12^#^	3.72 ± 0.17^##^
EE+Bai 200 EE+UDCA	241.75 ± 13.05 256.75 ± 19.31	10.35 ± 1.32^#^ 9.84 ± 4.16^#^	3.93 ± 0.09^#^ 3.76 ± 0.09^#^

The data are presented as mean ± SD. (n = 6). *p < 0.05 and **p < 0.01 versus control group, ^#^p < 0.05 and ^##^p < 0.01 versus EE group.

To determine structural changes in liver and hepatocytes by histology, liver sections were stained with H&E and TEM respectively. H&E-stained liver sections showed that hepatic cells in EE-treated rats were disordered in arrangement, and showed cell nuclear pyknosis, edema, widening of intercellular spaces, and inflammatory infiltration (**Figure 1C**). However, liver injury was significantly alleviated in EE with baicalin 100, 200 mg/kg, and UDCA groups (**Figure 1D**). Moreover, we also observed microstructural changes in the liver by TEM. As shown in **Figure 1D**, hepatocytes in the EE group showed disordered cell organelles, loose mitochondrial edema, decreased cytoplasmic vacuoles, and reduced microvilli in the capillary bile duct. After treatment with baicalin, these changes were reversed, which was especially true in the high-dose group. In UDCA-treated rats, pathological damage in the liver and microstructural changes was minor.

Taken together, these results were consistent with the findings in our previous report (Zhang CL et al., 2018) and demonstrated that baicalin had remarkable protective effects against EE-induced liver injury in rats.

### Baicalin Ameliorated EE-Induced Cholestasis and Restored the BAs Composition in Cholestasis Rats Liver

**Figure 2:**
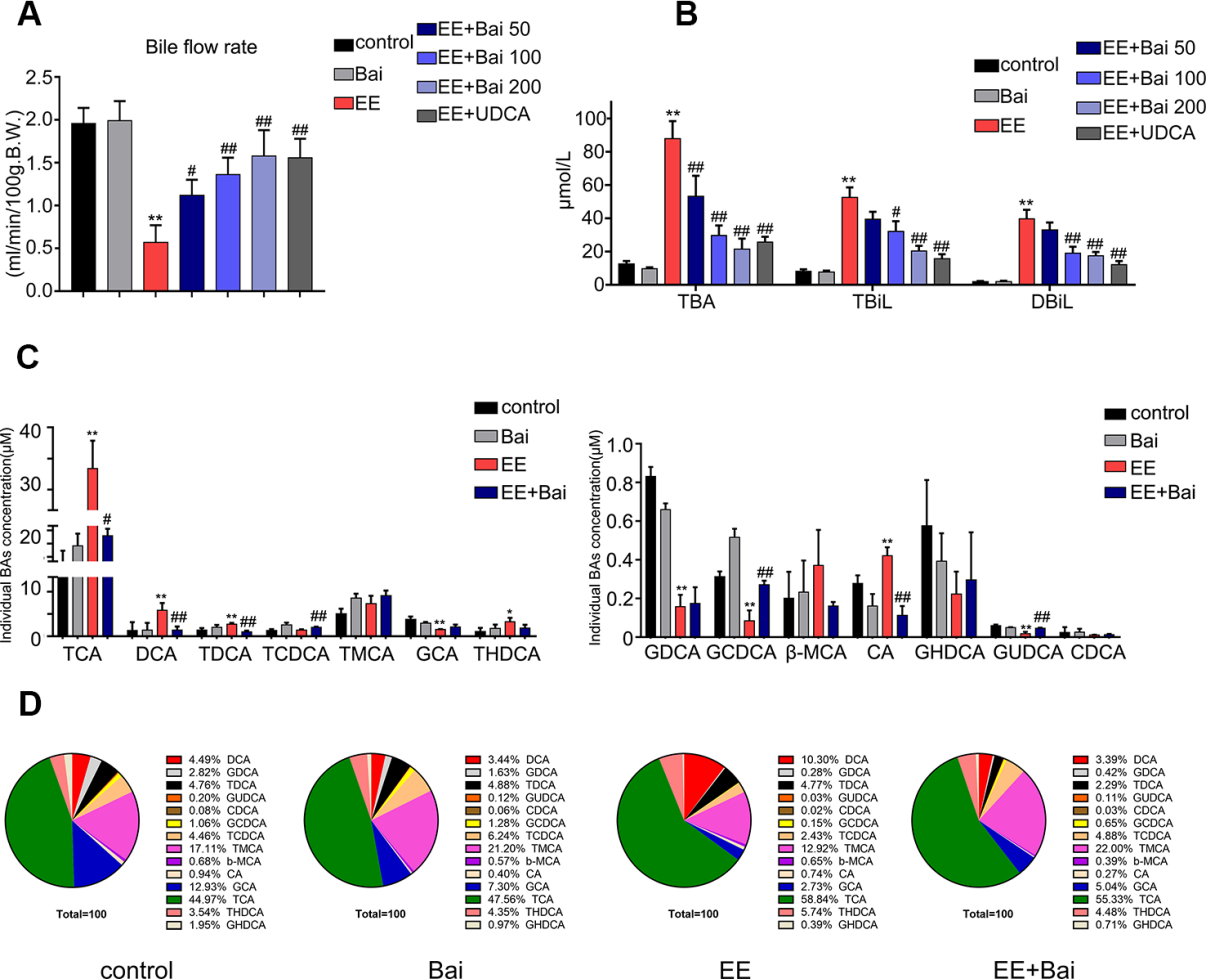
Baicalin attenuates EE-induced cholestasis and hepatic bile acid (BA) composition in rats. **(A)** The bile flow rate in rats over 2 hours. **(B)** Serum total bile acid (TBA), total bilirubin (**TBIL**), and direct bilirubin (**DBIL**). **(C)** Concentrations of hepatic individual BA in rats were determined by LC-MS/MS. Abbreviations: TCA, taurocholic acid; DCA, deoxycholic acid; TDCA, taurodeoxycholic acid; TCDCA, taurochenodeoxycholic acid; T-MCA, tauromuricholic acid; GCA, glycocholic acid; THDCA, taurohyocholic acid; GDCA, glycodeoxycholic acid; GCDCA, glycochenodeoxycholic acid; β-MCA, β-muricholic acid; CA, cholic acid; GHDCA, glycohyocholic acid; GUDCA, glycoursodesoxycholic acid; CDCA, chenodeoxycholic acid. **(D)** Hepatic BA composition in rats. Data are presented as the mean ± SD (n = 6). *p < 0.05 and **p < 0.01 versus the control group, ^#^p < 0.05 and ^##^p < 0.01 versus the EE group.

In this study, the bile flow was measured in rats. As reported, there was a remarkable bile flow obstruction in EE-treated rats (Meng et al., 2015). However, baicalin dose-dependently increased the EE-suppressed bile flow (**Figure 2A**). In addition, serum levels of TBA, TBiL, and DBiL were obviously increased in EE-induced cholestatic rats, and baicalin administration strikingly reduced these indexes (**Figure 2B**). Taken together, baicalin at a dose of 200 mg/kg had the best effect in EIC rats. Therefore, 200 mg/kg baicalin was used for further studies.

The composition and concentration of liver BA are closely related to liver damage (Woolbright et al., 2014). Therefore, we analyzed the BA composition in rat liver using a sensitive LC-MS/MS method as previously reported (Xiang et al., 2019). Representative chromatograms are shown in **Supplementary Figure 1**. In EE-treated rats, the hepatic concentration of TCA, DCA, TDCA, THDCA, and CA was remarkably increased. However, levels of GCA, GDCA, GCDCA, and GUDCA were significantly decreased (**Figure 2C**). Baicalin obviously increased the concentration of TCDCA, GCDCA, and GUDCA, and reduced levels of TCA, DCA, TDCA, and CA when compared with rats in the EE group (**Figure 2C**).

The BA composition in the liver was significantly different in the EE model group when compared with the control group. After administration of baicalin, the BA composition was nearly restored to control levels (**Figure 2D**).

### Baicalin Regulated the Expression of Enzymes Involved in BAs Synthesis and Detoxification

**Figure 3:**
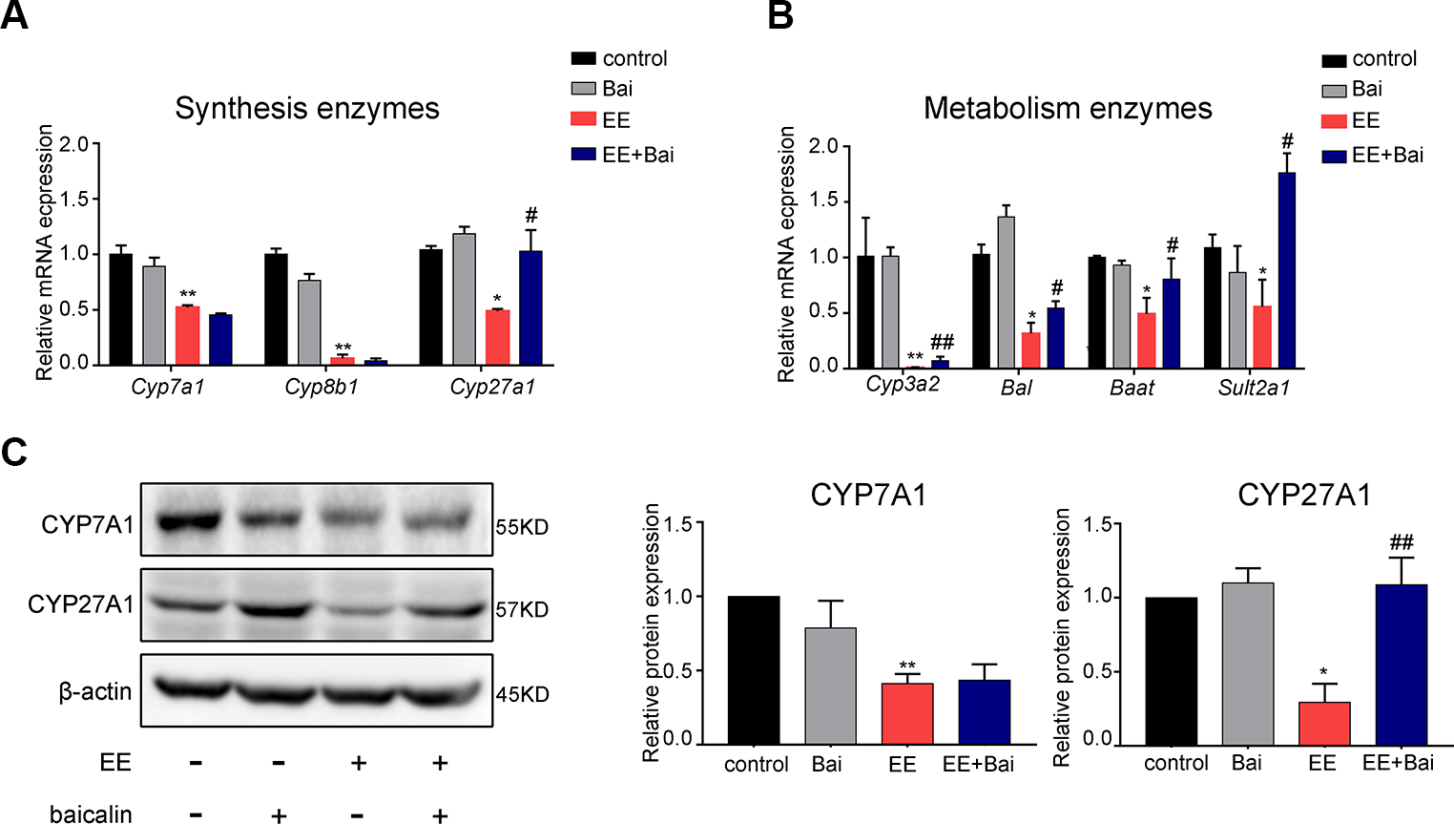
Baicalin alters the gene expression of hepatic enzymes involved in bile acid (BA) metabolism in rats. **(A)** The mRNA expression of BA synthetic enzymes, *Cyp7a1*, *Cyp8b1*, and *Cyp27A1*. **(B)** The mRNA expression of BA metabolism enzymes, *Cyp3a2*, *Bal*, *Baat*, and *Sult2a1*. **(C)** The protein levels of CYP7A1 and CYP27A1. Data are presented as the mean ± SD (n = 6). *p < 0.05 and **p < 0.01 versus the control group, ^#^p < 0.05 and ^##^p < 0.01 versus the EE group.

To explore the underlying mechanism involved in the hepatoprotective effects of baicalin, we examined the expression level of enzymes involved in BA synthesis and metabolism in the liver. In our study, EE significantly decreased mRNA levels of *Cyp7a1*, *Cyp8b1*, *Cyp27a1*, *Cyp3a2*, *Bal*, *Baat*, and *Sult2a1*, which were responsible for BA synthesis and metabolism, respectively. Nevertheless, baicalin treatment induced the gene expression of *Cyp27a1*, *Cyp3a2*, *Bal*, *Baat*, and *Sult2a1*, but had no significant effect on gene expression levels of *Cyp7a1* and *Cyp8b1* (**Figures 3A, B**). Moreover, we determined the protein expression levels of CYP7A1 and CYP27A1, which respectively are the key enzymes for the classic and alternative biosynthetic pathways of BA. As shown in **Figure 3C**, protein levels of CYP7A1 and CYP27A1 were decreased after EE administration. Baicalin treatment significantly induced protein levels of CYP27A1 but not of CYP7A1, which was consistent with the gene expression results.

Together, these findings suggested that baicalin partially restored BA synthesis inhibited by EE through inducing CYP27A1 expression, and promoting BA metabolism by increasing gene expression of *Cyp3a2*, *Bal*, *Baat*, and *Sult2a1*.

### Baicalin Reversed the Expression of the Hepatic Transporters

**Figure 4:**
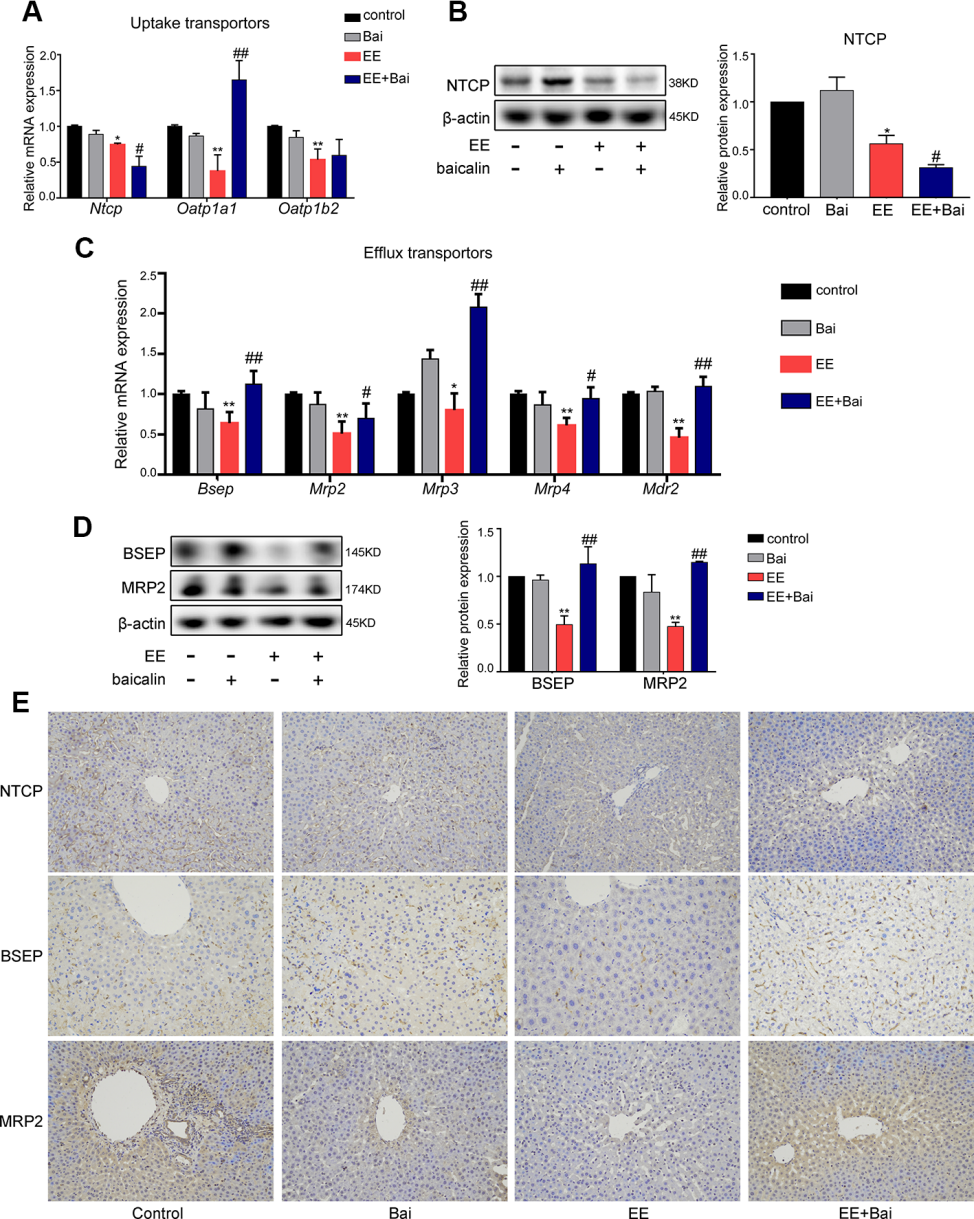
Baicalin promotes bile acid (BA) efflux and reduces BA influx in cholestatic rats. **(A)** The mRNA expression of BA uptake transporters, *Ntcp*, *Oatp1a1*, and *Oatp1b2*. **(B)** The protein level of NTCP. **(C)** The mRNA expression of BA efflux transporters, *Bsep*, *Mrp2*, *Mrp3*, *Mrp4*, and *Mdr2*. **(D)** The protein level of BSEP and MRP2. **(E)** Immunohistochemically staining for NTCP, BSEP, and MRP2 in rat liver. Data are presented as the mean ± SD (n = 6). *p < 0.05 and **p < 0.01 versus the control group, ^#^p < 0.05 and ^##^p < 0.01 versus the EE group.

BA transporters play important roles in BA homeostasis (Crocenzi et al., 2012). In this study, we measured the expression of hepatic BA transporters in rat liver. EE treatment significantly decreased gene expression levels of sinusoidal transporters, *Ntcp*, *Oatp1a1*, and *Oatp1b2* (**Figure 4A**) and *Mrp4* (**Figure 4C**), as well as canalicular transporters, *Bsep*, *Mrp2*, *Mrp3*, and *Mdr2* (**Figure 4C**). Baicalin treatment significantly increased mRNA expression of *Oatp1a1*, *Bsep*, *Mrp2*, *Mrp3*, *Mrp4*, and *Mdr2*. Interestingly, *Ntcp* mRNA was markedly decreased after treatment with baicalin (**Figure 4A**). To confirm these results, we determined the protein expression levels of NTCP, BSEP, and MRP2, which are key transporters involved in BA uptake and efflux (Chen et al., 2013). **Figures 4B**, **D** showed that proteins levels of NTCP, BSEP, and MRP2 were decreased by EE treatment and significantly increased after baicalin administration, which was in accordance with the gene expressions. In addition, immunohistochemical staining also demonstrated that the reduced expression of NTCP and increased expression of BSEP and MRP2 in EE-induced rats after baicalin treatment (**Figure 4E**; **Supplementary Figure 3**).

Together, these results suggested that baicalin protected against EE-induced liver injury due to a decrease in influx, and an increase in efflux of BA in the liver.

### Baicalin Regulated the Sirt1/HNF-1α/FXR Mediated Pathway in EIC Rats

**Figure 5:**
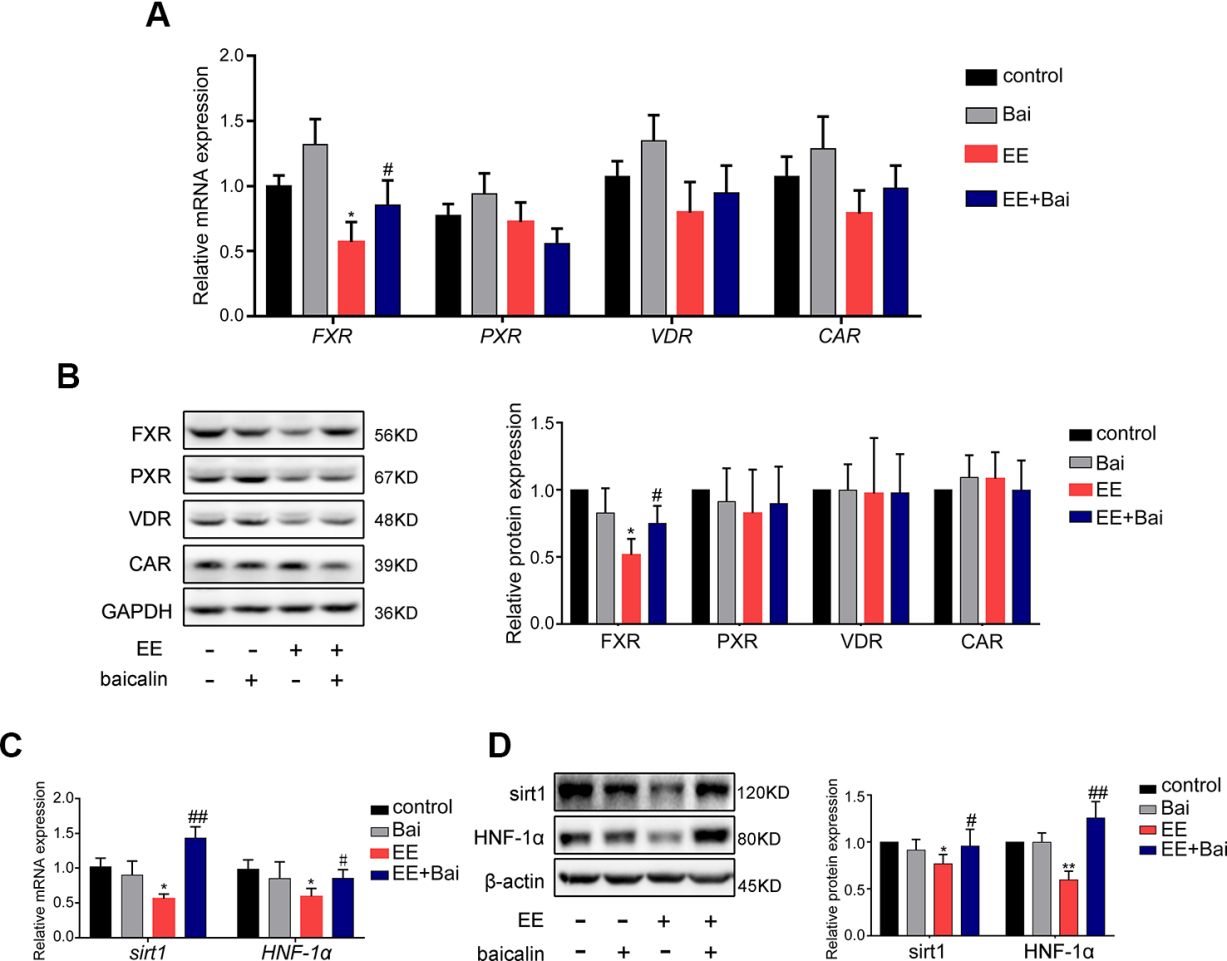
Baicalin restored the mRNA and protein level of FXR, Sirt1, HNF-1α *in vivo*. **(A)** The mRNA expression of nuclear receptors of *FXR*, *PXR*, *VDR*, and *CAR*. **(B)** The protein level of FXR, PXR, VDR, and CAR. **(C)** The mRNA expression of *Sirt1* and *HNF-1α*. **(D)** The protein level of Sirt1 and HNF-1α. Data are presented as the mean ± SD (n = 6). ^*^p < 0.05 and ^**^p < 0.01 versus the control group, ^#^p < 0.05 and ^##^p < 0.01 versus the EE group.

To investigate the mechanism underlying the amelioration of EE-induced hepatotoxicity and cholestasis after baicalin treatment, we focused on hepatic nuclear receptors: FXR, CAR, PXR, and VDR that are closely related to BA homeostasis (Gonzalez-Sanchez et al., 2015). In our study, we showed that both mRNA and protein levels of FXR were significantly decreased in the EE-treated group. Interestingly, baicalin administration completely reversed this effect (**Figures 5A, B**). No significant differences were observed in the expression of CAR, PXR, and VDR (**Figures 5A, B**).

Furthermore, we determined the expression of Sirt1 and HNF-1α, which are genes upstream of FXR. In previous studies, it has been revealed that Sirt1/HNF-1α plays an important role in maintaining BA homeostasis by regulating FXR expression (Purushotham et al., 2012; Kazgan et al., 2014; Yu et al., 2016). In this study, we found that the expression of genes and proteins of Sirt1 and HNF-1α were significantly inhibited by EE treatment (**Figures 5C, D**), which was consistent with the findings published by Yu et al. (Yu et al., 2016). Thus, baicalin significantly reversed the expression of Sirt1 and HNF-1α both at the gene and protein level.

### Baicalin Activates the Sirt1/HNF1α/FXR Signaling Pathway *In Vitro*

**Figure 6:**
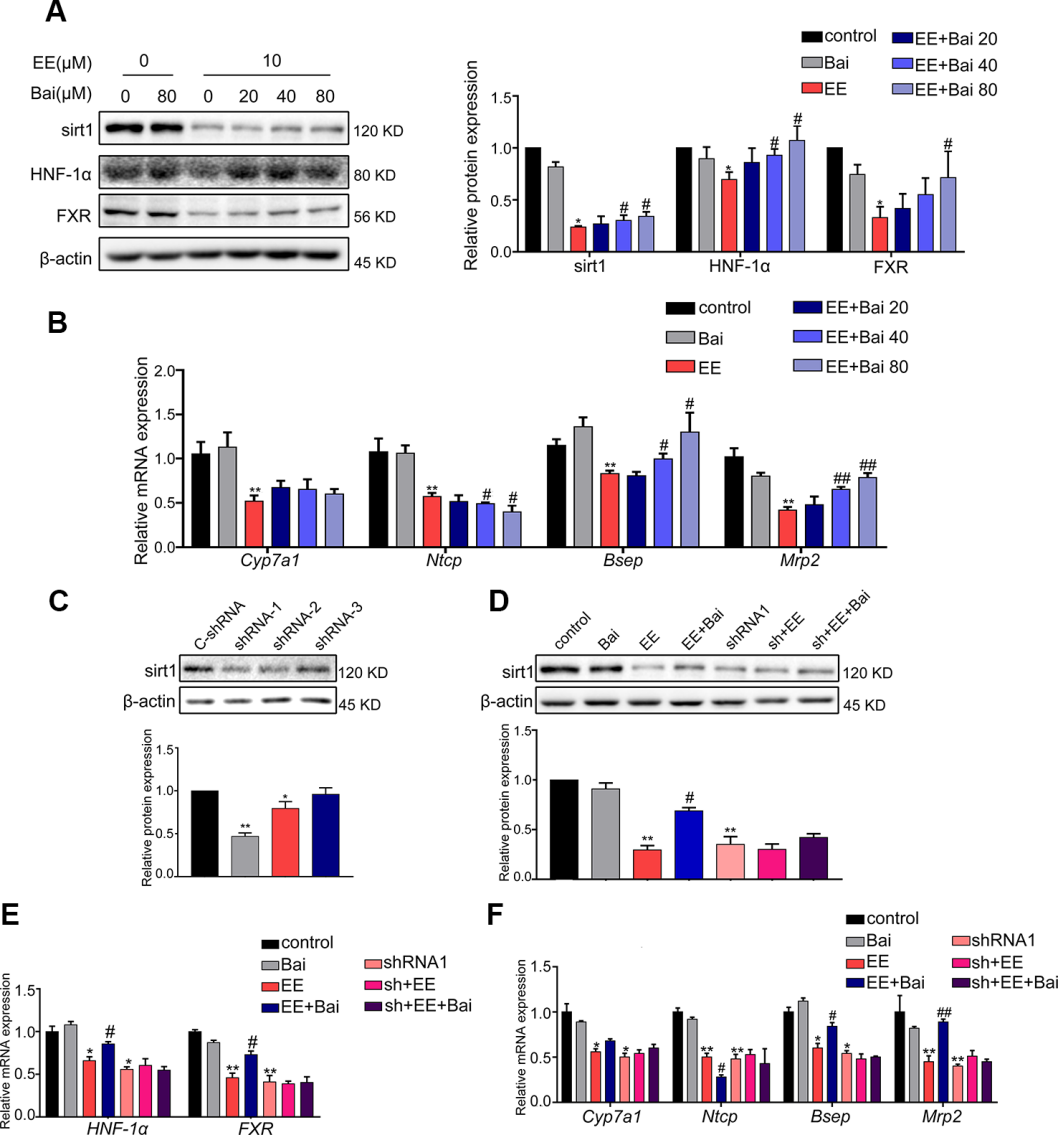
Effect of baicalin on Sirt1, HNF-1α, FXR, and FXR target genes *in vitro*. **(A)** HepG2 cells were treated with EE (10 μM), and baicalin (20, 40, 80 μM), and incubated with EE for 24 hours. EE inhibited the protein expression of Sirt1, HNF-1α, and FXR, which was reversed by baicalin treatment in a dose-dependent manner. **(B)** The mRNA expression of *Cyp7a1*, *Ntcp*, *Bsep*, and *Mrp2* in HepG2 cells treated with EE (10 μM), and baicalin (20, 40, 80 μM) for 24 hours. **(C)** The protein level of Sirt1 in HepG2 cells, which were transduced with shRNA targeting Sirt1 (shRNA 1-3) or negative control (C-shRNA) for 72 hours. **(D)** The protein expression of Sirt1 in HepG2 cells treated with EE (10 μM), baicalin (80 μM), EE (10 μM) with baicalin (80 μM), and shRNA1. **(E)** The mRNA expression of HNF-1α and FXR in HepG2 cells treated with EE (10 μM), baicalin (80 μM), and shRNA1. **(F)** The mRNA expression of *Cyp7a1*, *Ntcp*, *Bsep*, and *Mrp2* in HepG2 cells treated with EE (10 μM), baicalin (80 μM), and shRNA1. Data are presented as the mean ± SD (n = 3). ^*^p < 0.05 and ^**^p < 0.01 versus the control group, ^#^p < 0.05 and ^##^p < 0.01 versus the EE group.

In this study, the effect of baicalin on Sirt1/HNF-1α/FXR activation was examined in HepG2 cells. As shown in **Figure 6A**, EE completely inhibited the protein expression of Sirt1, HNF-1α, and FXR. However, this inhibition was restored by baicalin treatment in a dose-dependent manner. Next, we determined the expression of *Cyp7a1*, *Ntcp*, *Bsep*, and *Mrp2*, which are target genes of FXR. Treatment with baicalin up-regulated the gene expression of *Bsep* and *Mrp2*, and repressed *Ntcp* gene expression (**Figure 6B**). These results were consistent with the above *in vivo* findings.

To further verify the crucial role of Sirt1 in EE-induced cholestasis, specific shRNA plasmids were constructed to down-regulate the expression of Sirt1 in HepG2 cells. The constructed Sirt1 shRNA1 could efficiency down-regulate the expression of Sirt1 (**Figure 6C**). The baicalin-induced increase in Sirt1 compared with that of the EE-treated group was completely blocked by Sirt1 shRNA1 (**Figure 6D**). Furthermore, the baicalin-induced increase of FXR, HNF-1α, and FXR target genes were also reversed after down-regulating the expression of Sirt1 (**Figures 6E, F**). These findings further demonstrated that baicalin-induced activation of Sirt1/HNF-1α/FXR was responsible for improving EE-induced hepatotoxicity.

### Baicalin Inhibits Inflammatory Responses in the Livers of EIC Rats

**Figure 7:**
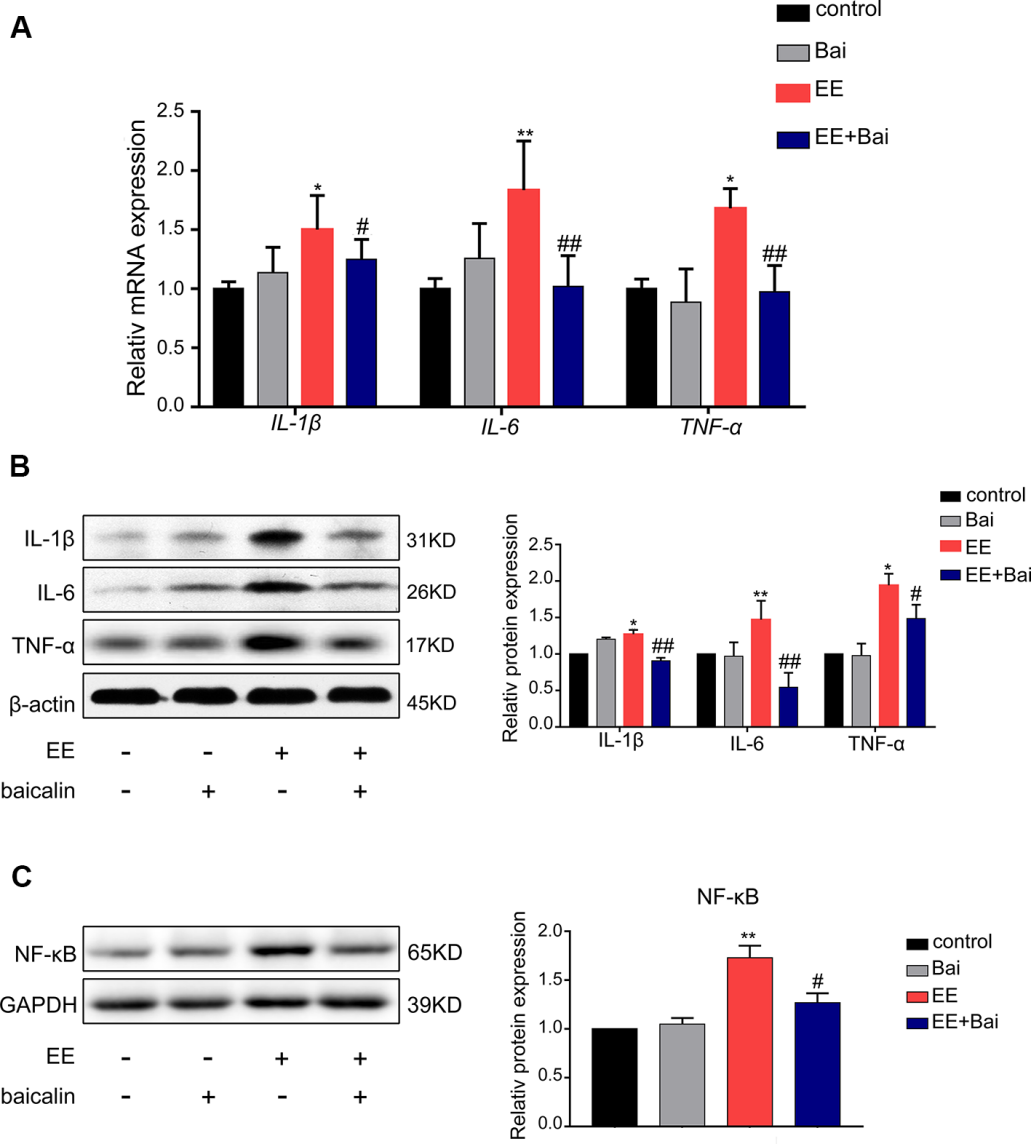
Baicalin decreased the level of inflammatory factors through the NF-κB pathway in the liver. **(A)** The mRNA level of *IL-1β*, *IL-6*, and *TNF-α* in rat liver. **(B)** The protein expression of IL-1β, IL-6, and TNF-α in rat liver. **(C)** The protein expression of NF-κB in rat liver. Data are presented as the mean ± SD (n = 6). *p < 0.05 and **p < 0.01 versus the control group, ^#^p < 0.05 and ^##^p < 0.01 versus the EE group.

The inflammatory response is one of the characteristics of cholestasis. Therefore, we determined the mRNA and protein expression level of IL-6, IL-1β, and TNF-1α. As shown in **Figures 7A, B**, both the mRNA and protein expression of IL-6, IL-1β, and TNF-1α were significantly up-regulated in EE-treated group compared to the control group, but were statistically reversed in baicalin-treated rats. To further explore the anti-inflammatory mechanism of baicalin in cholestasis rats, we examined the protein expression level of NF-κB, which is considered a “master switch” in the regulatory mechanism of inflammatory processes that can regulate numerous inflammatory factors, including IL-6, IL-1β, and TNF-1α (Liu et al., 2017; Zhang J et al., 2018). As shown in **Figure 7C**, In the EE-treated group, the expression level of NF-kB was significantly increased; however baicalin significantly reversed this effect. These findings may suggest that baicalin is responsible for reducing inflammation in EE-induced cholestasis *via* the NF-kB pathway by down-regulating IL-6, IL-1β, and TNF-1α expression.

## Discussion

The abnormal increase in estrogens and their metabolites can easily lead to cholestasis, which results in the impairment of bile flow and hepatotoxicity (Trauner et al., 1998). Baicalin is the main flavonoid component isolated from the traditional Chinese medicine *Scutellaria baicalensis* (You et al., 2018). In previous studies, it has been revealed that baicalin possess effective therapeutic effects on liver diseases (Guo et al., 2009; Dai et al., 2018; Xu et al., 2018). In our previous study, we demonstrated that baicalin ameliorated liver injury in EIC rats; however the underlying mechanisms remained to be elucidated (Zhang CL et al., 2018). In the current study, we showed that baicalin protected against EE-induced cholestasis by ameliorating liver histology, decreasing serum biomarkers, as well as increasing bile flow and reducing BA accumulation in the liver (**Figures 1** and **2**). Importantly, our results demonstrated that the main role of baicalin on improving cholestatic liver injury was to restore BA homeostasis through activating the Sirt1/HNF-1α/FXR signaling pathway. By the way, we found that baicalin itself does not alter the expression of enzymes, transporters and nuclear receptors, but reverse these changes in EE-induced rat liver through activating sirt1 signaling pathway. This phenomenon was also present in SRT1720, an activator of sirt1, which also had a hepatoprotective effect on cholestatic liver injury by activating sirt1 (Kulkarni et al, 2016).

BA is the main component of bile, and known to stimulate bile flow and secretion from the liver (Zhou and Hylemon, 2014). In addition, BAs also plays important roles in regulating hepatic metabolism and drug disposition as signaling molecules (Vítek and Haluzík, 2016). In previous reports using animal models, it was shown that impairment of bile formation and secretion was associated with the pathogenesis of EIC (Chen et al., 2013). Furthermore, an abnormal BA composition and accumulation of BA in the liver can cause cholestatic liver injury in EIC animals, as well as in EIC patients (Sinakos and Lindor, 2010; Xiang et al., 2019). In this study, we analyzed the composition and concentration of individual BAs in the liver. Our results showed that the concentration of CA and TCA was significantly increased in EE-treated rat liver. Song et al. demonstrated that the increase in BA aggravated liver injury, among which CA and TCA might be the most sensitive indicators in alpha-naphthylisothiocyanate (ANIT)-induced cholestatic liver injury (Lin et al., 2019). GCA, GDCA, and GCDCA, which are more hydrophilic and less toxic, were markedly decreased (**Figure 2C**). However, this phenomenon was changed by baicalin treatment. Moreover, baicalin treatment also restored abnormal BA composition in EIC rats (**Figure 2D**).

Numerous studies have revealed that BA homeostasis was tightly related to the process of BA synthesis, metabolism, and transport (Trauner et al., 2017). In the present study, we found baicalin had several roles in regulating BA homeostasis in rat liver. First, we found baicalin restored the expression of BA synthesis (CYP27A1) and metabolism enzymes (Cyp3a2, Bal, Baat, and Sult2a1), which were inhibited by EE. BA synthesis undergoes two pathways: the classis pathway and the alternative pathway. In humans, the classic pathway accounts for 90% total BA synthesis and is catalyzes by CYP7A1 and CYP8B1 (Li and Chiang, 2013). The alternative pathway is initiated by CYP27A1 and contributes to only 10% of BA synthesis in the liver. In our results, EE significantly inhibited the expression of CYP7A1 and CYP8b1, while baicalin further mildly downregulated the expression of CYP7A1 and CYP8B1 (**Figure 3A**). Overall, baicalin may not aggregate BA synthesis. Moreover, the alternative pathway mainly produces CDCA, but our result showed that baicalin had no significance on the concentration of CDCA in the liver (**Figure 2C**). Therefore, baicalin could not increase the BA synthesis. By the way, baiclain significantly inducing the expression of Cyp3a2, Bal, Baat, and Sult2a1, which are contributed for BA detoxification. Second, Baicalin could inhibit hepatic BA uptake by downregulated the expression of NTCP. In human, enterohepatic circulation of BA is highly efficient in human; most BAs (95%) are reabsorbed into feces and are replenished by *de novo* synthesis. NTCP located in the sinusoidal membrane, which is responsible for >80% of conjugated BAs uptake into the liver cells (Lu et al, 2019), thus is the most important uptake transporters in the liver (Li and Chiang, 2013). Slijepcevic et al. demonstrated that NTCP expression is reduced during cholestasis, but the further pharmacological inhibition still efficiently reduced hepatocellular accumulation of bile salts and had protective effects on cholestatic liver injury (Slijepcevic et al, 2018). In contrast to NTCP, our results showed baicalin increased the expression of Otp1a1 (**Figure 4A**), which belongs to Oatps family. Oatps are multispecific transporter systems with a wide substrate preference for mostly amphipathic organic compounds, including conjugated and unconjugated bile salts, bromosulfophthalein (BSP), bilirubin, and so on, and the BA uptake by Oatps is quantitatively less significant than NTCP (Trauner and Boyer, 2003). In addition, there are three members of Oatps family in rat liver (Jetter and Kullak-Ublick, 2019). Thus, the role of Otp1a1 in BA uptake is far less prominent. Third, baiclain significantly increased the expression of BSEP and MRP2, which are responsible for BA efflux in the liver. What’s more, the mRNA expression of *Mrp3* and *Mrp4* were induced after baicalin administration in the kidney for promoting the excretion of BA in EIC rats (**Supplementary Figure 2**). Thus, we suggested that baicalin alleviated EE-induced liver injury through maintaining BA homeostasis *via* regulating the synthesis, metabolism, and transport processes of BA.

It is well known that nuclear receptors, including PXR, VDR, CAR, and FXR, play a crucial role in modulating BA homeostasis (Lu et al., 2000; Li and Chiang, 2013). These nuclear receptors paly critical roles in regulating the expression or transcription of genes involved in BA synthesis, metabolism, and transport (Li and Chiang, 2013; Gonzalez-Sanchez et al., 2015). In this study, we demonstrated that mRNA and protein levels of hepatic nuclear receptors, including PXR, CAR, and VDR were not statistically different between cholestatic and baicalin-treated cholestatic rats (**Figure 5**). However, there was a significant down-regulation of FXR in EIC rats, and baicalin significantly restored the expression of FXR both in mRNA and protein levels (**Figure 5**). Milona et al. have shown that the expression of FXR was inhibited in mice during pregnancy, which resulted in increased expression of procholestatic genes, and increased hepatic BA levels (Milona et al., 2010). Furthermore, GW4064, a FXR agonist, has been demonstrated to protect against EIC in rat models (Liu et al., 2003). Thus, activating FXR may be a therapeutic strategy for EE-induced liver injury. It is well known that FXR acts as the primary sensor of BA and possesses master moderating effects in BA homeostasis (Kuipers et al., 2004). Both conjugated and unconjugated BAs can active FXR at physiological concentrations (Merk et al., 2014). BA-activated FXR reduced BA uptake by down-regulation of NTCP and increased BA excretion through BSEP, MRP2 induction, thereby protecting hepatocytes against hepatotoxic BA overload (Rizzo et al., 2005). FXR repressed CYP7A1 and CYP8B1 through the SHP/LRH-1 pathway for decreasing BA synthesis (Lu et al., 2000; Davis et al., 2002). In addition, CYP27A1, the rate-limiting enzyme in the alternative pathway of BA synthesis, also acts as a target gene of FXR (Zhang F et al., 2018). Moreover, activating of FXR by GW4064 down-regulated the expression of CYP27A (Rosales et al., 2013). In the present study, we showed that baicalin had hepatoprotective effects on EIC rats, thereby alleviating EE-induced liver injury as well as the suppression of BA synthetic enzymes. Further, CYP3A, CYP2B, SULT2A1, BAL, and BAAT, which convert BAs into more hydrophilic derivatives, are also positively regulated by FXR (Song et al., 2001; Xiang et al., 2019). Thus, our results demonstrated that baicalin protected against EE-induced liver injury by maintaining BA homeostasis in rat liver *via* restoring BA synthesis, inducing BA metabolism and efflux as well as repressing BA uptake through activating of the FXR pathway.

Sirt1, the most studied member of the family of sirtuins, is a critical regulatory factor in hepatic BA (Leibiger and Berggren, 2006; Lee and Kemper, 2010). In previous studies, it was shown that Sirt1 may increase HNF-1α DNA binding activity, and induce FXR gene transcription by upregulating HNF-1α (Kemper et al., 2013). Deficiency of Sirt1 in hepatocyte decreased binding of HNF-1α to the FXR promoter, thereby leading to derangements in BA metabolism, inducing the development of cholesterol gallstones in mice (Kazgan et al., 2014). Therefore, Sirt1/HNF-1α/FXR signaling plays a key role in maintaining systemic BA homeostasis, and can protect animals from cholestatic liver injury (Yu et al., 2016). In our study, we demonstrated that Sirt1 and HNF-1α were greatly reduced by EE treatment (**Figure 5**). After treatment with baicalin, the suppression of Sirt1/HNF-1α/FXR was reversed in a dose-dependent manner *in vitro*, which could be totally blocked after transfection with a specific sh-RNA targeting Sirt1 mRNA (**Figure 6**). The above findings demonstrated that baicalin improved hepatic BA homeostasis *via* the Sirt1/HNF-1α/FXR signaling pathway in EIC rats.

It has previously been reported that the accumulation of toxic BAs in hepatocytes leads to the overexpression of pro-inflammatory cytokines, thereby triggering liver inflammation, and promoting the development and progression of cholestasis (Yang et al., 2019). Inflammatory factors, including TNF-α, IL-6, and IL-12 can be upregulated by the activation of NF-κB, leading to aberrant leukocyte infiltration and inflammation in EIC patients (Zhang et al., 2016; Shao et al., 2017). Moreover, damage to mitochondria and the endoplasmic reticulum (ER) was observed in placenta with increased inflammatory cytokines including IL-1β, TNF-α, and regulating NF-kB pathway possesses protective effects in EIC rats (Han et al., 2018; Zhang Y et al., 2018). Our data showed that hepatic TNF-α, IL-1β, and IL-6 were significantly elevated in response to the increased expression of NF-κB in EE-treated rats (**Figure 7**). However, baicalin completely reversed this change, as well as the expression of TNF-α, IL-1β, and IL-6 (**Figure 7**). Therefore, our data suggested that baicalin might influence relieving EE-induced inflammatory by inhibiting NF-κB signaling. It has previously been reported that activation of Sirt1 decreased the production of several pro-inflammatory cytokines, including TNF-α, IL-12, and IL-18 in EIC rats and protected syncytiotrophoblasts against TCA-induced inflammatory injury through the Sirt1-NF-κB signaling pathway (Liao et al., 2018). Moreover, NF-κB is a downstream gene of FXR, and upregulating Sirt1/FXR/NF-κB signaling had an efficient protective effect on rats from hemorrhagic shock induced liver injury by decreasing inflammatory responses (Zhang et al., 2017; Sun et al., 2019; Yu et al., 2019). Thus, we hypothesized that activation of FXR by baicalin through the Sirt1/HNF-1α signaling pathway might also play a key role in regulating the NF-κB pathway, which would need to be further investigated.

**Figure 8:**
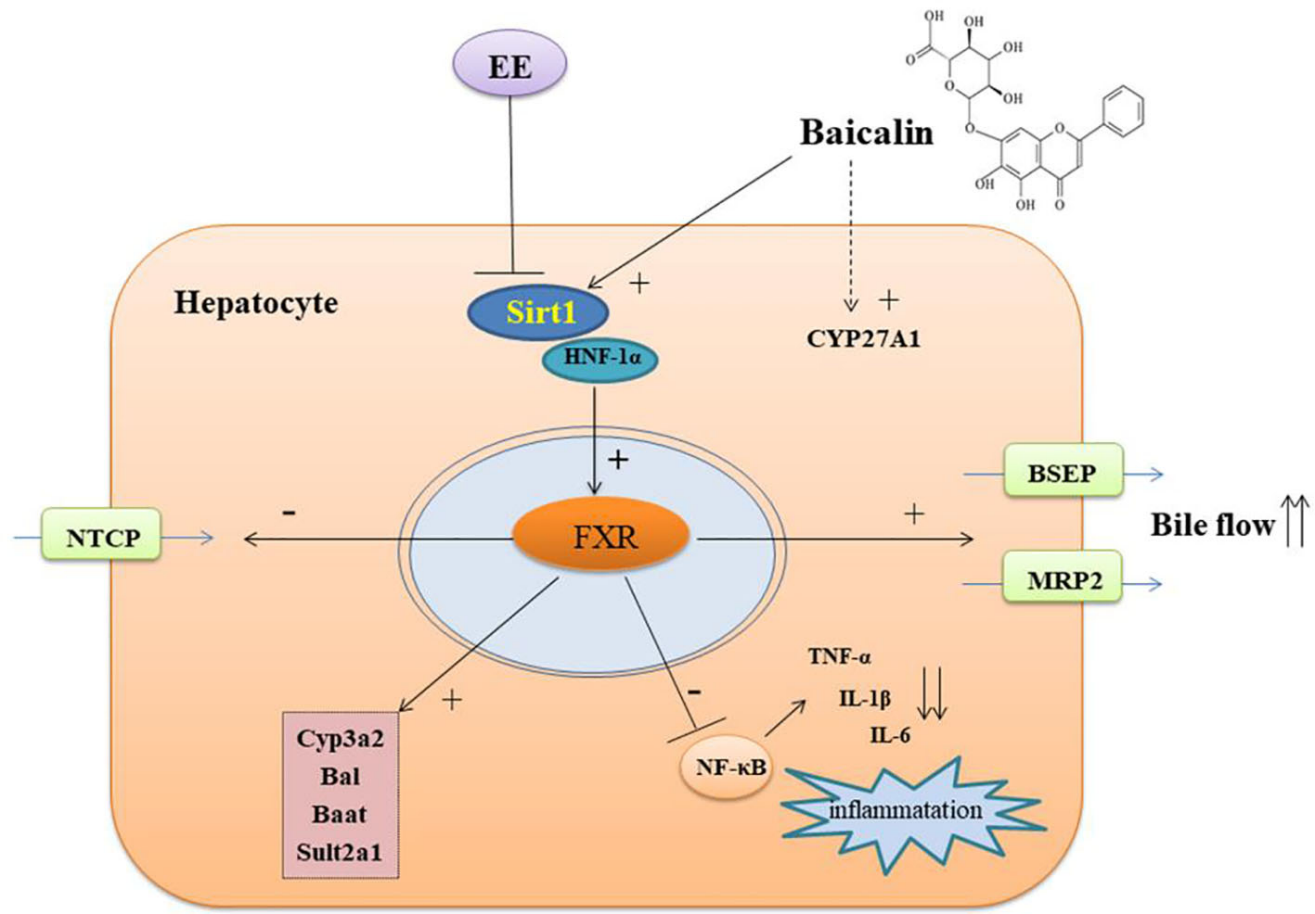
The possible mechanism of baicalin on protect against EE-induced cholestatic liver injury.

In summary, our results demonstrated that baicalin protected EIC rats against cholestatic liver injury by restoring hepatic BA homeostasis and modulating the inflammatory response through activating the Sirt1/HNF-1α/FXR signaling pathway (**Figure 8**). Thus, our findings suggested that baicalin can be used as a potential therapeutic drug for the treatment of cholestatic liver disease.

## Data Availability Statement

The datasets analyzed in this article are not publicly available. Requests to access the datasets should be directed to ld2069@outlook.com.

## Ethics Statement

The animal study was conducted in accordance with the National Institutes of Health guide for the care and use of laboratory animals. All animal studies were approved by the Ethics Committee on Animals Experimentation of Tongji Hospital (Wuhan, China).

## Author Contributions

JY designed the study, analyzed the data and wrote the manuscript. DaX contributed to data collection and performed the experiments. DoX was responsible for the idea. WH performed *in vivo* experiments. YL, LL, GL, and CJ contributed to the *in vitro* experiments. XR partly analyzed the data. DL and CZ supervised the study. All authors reviewed and approved the manuscript.

## Funding

This work was supported by the National Natural Science Foundation of China (Nos. 81670521 and 81573788).

## Conflict of Interest

The authors declare that the research was conducted in the absence of any commercial or financial relationships that could be construed as a potential conflict of interest.
