# Supplementary material for: Baicalin Protects Against 17α-Ethinylestradiol-Induced Cholestasis via the Sirtuin 1/Hepatic Nuclear Receptor-1α/Farnesoid X Receptor Pathway
Source: Front Pharmacol. 2020 Feb 11;10:1685. doi: 10.3389/fphar.2019.01685 (PMC7026019; doi:10.3389/fphar.2019.01685)
Supplement: Supplementary file 1 [file DataSheet_1.pdf]

Supplementary Materials

Baicalin protects against 17α-ethinylestradiol-induced cholestasis via the Sirt1/HNF-1α/FXR pathway

Jinyu yang [1], Daochun Xiang [1,2], Dong Xiang [1], Wenxi He [1], Yanan Liu [1], Lulu Lan [1], Guodong Li [1], Chen Jiang [3], Xiuhua Ren [1], Dong Liu [1]*, Chengliang Zhang [1]*

*** Correspondence:**

Dong Liu, ld2069@outlook.com;

Chengliang Zhang, clzhang@tjh.tjmu.edu.cn.

1. Supplementary figures

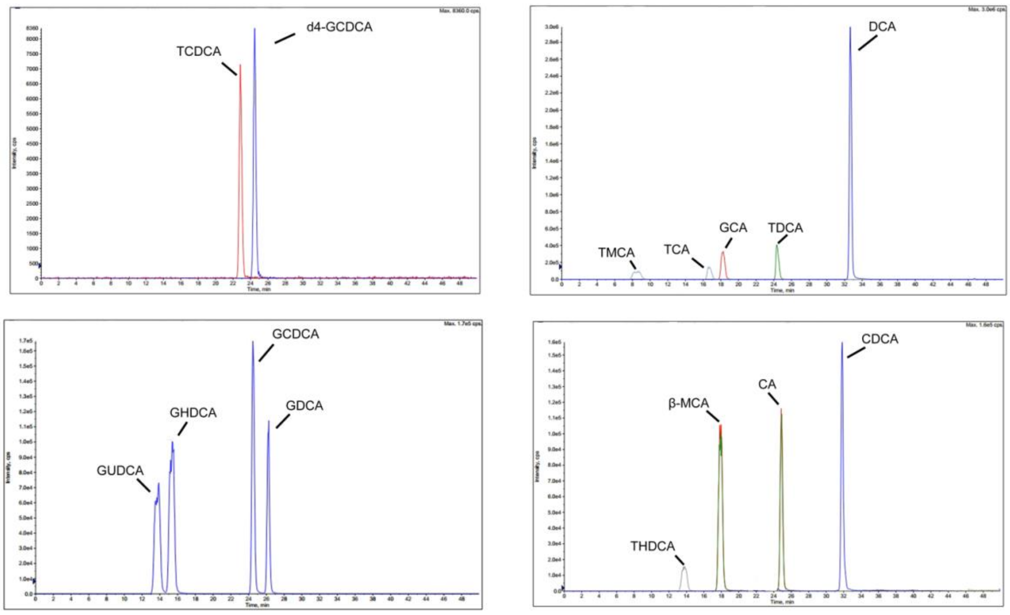

Supplementary figure 1 Representative LC-MS/MS chromatogram of bile acids in liver. Abbreviations: TCA, taurocholic acid; DCA, deoxycholic acid; TDCA, taurodeoxycholic acid; TCDCA, taurochenodeoxycholic acid; T-MCA,

tauromuricholic acid; GCA, glycocholic acid; THDCA, taurohyocholic acid; GDCA, glycodeoxycholic acid; GCDCA, glycochenodeoxycholic acid; β-MCA, β-muricholic acid; CA, cholic acid; GHDCA, glycohyocholic acid; GUDCA, glycoursodesoxycholic acid; CDCA, chenodeoxycholic acid; d4-glycochenodeoxycholic acid, d4-GCDCA.

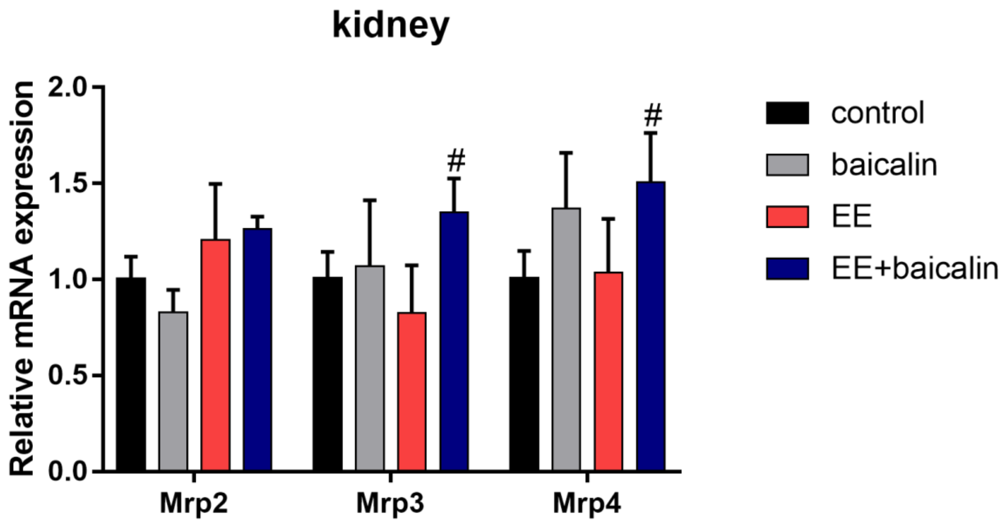

Supplementary figure 2 The mRNA expression of Mrp2, Mrp3 and Mrp4 in the rat kidney. Data are presented as the mean ± SD (n=6), Significant differences compared with EE group, $^{\#}p<0.05$.

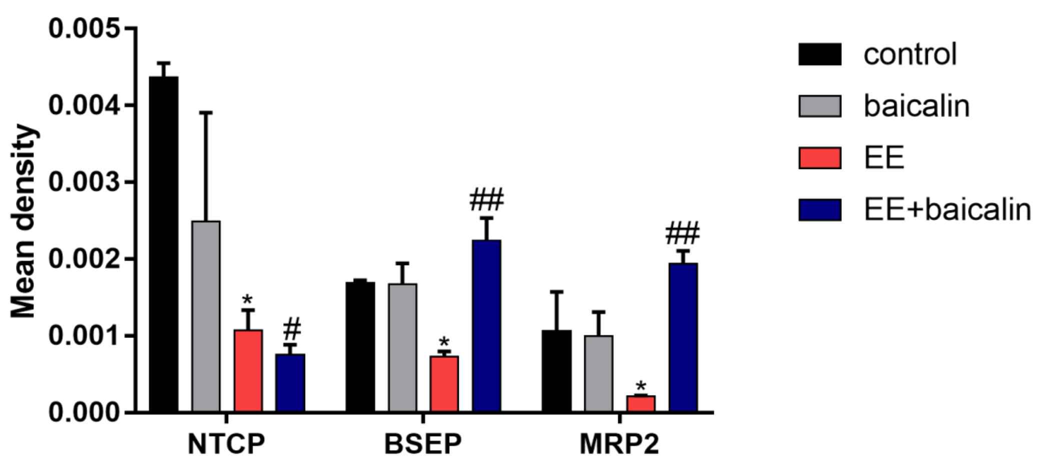

Supplementary figure 3 The mean density of hepatic NTCP, BSEP and MRP2 using immunohistochemically staining in rat liver. At least three 200x fields of view were randomly selected for each slice in each group for photographing. When taking pictures, try to fill the entire field of vision with the organization, and ensure that the background light of each photo is consistent. Analyze each picture to obtain the cumulative optical density value (IOD) and the pixel area of the tissue (AREA) for each photo. And then analyze the average optical density value IOD / AREA (Mean Density). Data are presented as the mean ±SD (n=3). *p<0.05 versus the control group, #p<0.05 and ##p<0.01 versus the EE group.

2. Supplementary tables

Supplementary Table 1 The primer sequences used for real-time PCR for rats.

Gene	Forward primer	Reverse primer
FXR	TGAGCGTCTACAGCGAAAGTG	GGGATGGTGGTCTTCAAATAAG
CAR	ACCAGTTTGTGCAGTTCAGG	CTTGAGAAGGGAGATCTGGT
PXR	GACGGCAGCATCTGGAACTAC	TGATGACGCCCTTGAACATG
VDR	GCCCCTCATAAAGTTCCAGGTG	GGATAGGCGGTCCTGAATGG
Sirt1	TGTTTCCTGTGGGATACCTGA	TGAAGAATGGTCTTGGGTCTTT
HNF-1α	AAGATGACACGGATGACGATG	TGTTGAGGTGCTGGGACAGG

	G	
Ntcp	TGCACCATAGGGATCGTCCTC	GATGCTGTTGCCCACATTGA
Oatp1a1	TGGGGAAGGTTGCTGGCCCAATTT	GGTGGTTAATCCAGCAACTGCTGC
Oatp1b2	AGACGTTCCCATCACAACCAC	GCCTCTGCAGCTTTCCTTGA
Bsep	CGTGCTTGTGGAAGAAGTTG	GGGAGTAGATGGGTGTGACTG
Mrp2	CTCGGTCTTATGCGGCGTATTC	CCGTGACTGATGGAGTTTGTGTT
Mrp3	TCCCACTTCTCGGAGACAGTAAC	CTTAGCATCACTGAGGACCTTGAA
Mrp4	GAAGGAAAATGAGGAAGCAGAG	GGATGACTGTTGAGACCAAATC
Mdr2	CTGTAGCGGGAATTGTTG	GATGCCGTAGATGTGAGC
Cyp7a1	GTCCGGATATTCAAGGATGC	GGGAATGCCATTTACTTGGA
Cyp8ba1	ATGAAGGCTGTGCGAGAG	TCTCTTCCATCACGCTGTC
Cyp27a1	GGAAGGTGCCCCAGAACAA	GCGCAGGGTCTCCTTAATCA
Cyp3a2	TGACTGCTCTTGATGCATGGTT	ATCACAGACCTTGCCAACTCCT
Sult2a1	AGGAACGAACTGGCTGATTG	ATGGGAAGATGGGAGGTCAT
Bal	TGCCCTTGCTACACTCTG	TGGTCCCTGAAGTATAGATG
Batt	CTGTCGAACTACGGTTTTGGCGAA	TCAGGCCTGTGACCCGGATA

Il-6	GGATACCACCCACAACAGACC	ACGGAACTCCAGAAGACCAG<br>A
Tnf-a	CAGACCCTCACACTCAGATCA<br>TCTT	CAGAGCAATGACTCCAAAGTA<br>GACCT
Il-1β	TTCTTTTCCTTCATCTTTGAAG<br>AAG	TCCATCTTCTTCTTTGGGTATT<br>GTT

Supplementary Table 2 The primer sequences used for real-time PCR for human.

Gene	Forward primer	Reverse primer
FXR	GACTTTGGACCATGAAGACCA<br>G	GCCCAGACGGAAGTTTCTTAT<br>T
Sirt1	TAGCCTTGTCAGATAAGGAAG<br>GA	ACAGCTTCACAGTCAACTTTG<br>T
HNF-1α	GGTCCTACGTTCACCAACACA	CTCTGGGTCACATGGCTCT
Ntcp	AAGGACAAGGTGCCCTATAAA<br>GG	TTGAGGACGATCCCTATGGTG
Bsep	GCCGCAGCTCGTCAGATAC	GAATTGCAGTCAAACCACCCT<br>AT
Mrp2	TCTCTCGATACTCTGTGGCAC	CTGGAATCCGTAGGAGATGAA<br>GA
Cyp7a1	AGAAGCATTGACCCGATGGAT	AGCGGTCTTTGAGTTAGAGGA
